# Hormonal Profile and Anthropometric Indices As Determinants of Metabolic Risk in Polycystic Ovary Syndrome: A Prospective Observational Study

**DOI:** 10.7759/cureus.105995

**Published:** 2026-03-27

**Authors:** Nigil Napolean, Shraddha Chaurasiya, Soumya Khanna, Shikha Sachan, Royana Singh, Jhumur Dey

**Affiliations:** 1 Anatomy, Institute of Medical Sciences, Banaras Hindu University, Varanasi, IND; 2 Obstetrics and Gynaecology, Institute of Medical Sciences, Banaras Hindu University, Varanasi, IND; 3 Electronic and System Engineering, Indian Institute of Science, Bengaluru, IND

**Keywords:** anthropometric indices, hormonal imbalance, insulin resistance, metabolic dysfunction, polycystic ovary syndrome

## Abstract

Background

Polycystic ovary syndrome (PCOS) is a heterogeneous endocrine-metabolic disorder characterized by reproductive, hormonal, and metabolic disturbances. This study aimed to evaluate the association between clinical features, anthropometric indices, hormonal parameters, metabolic profile, ultrasonographic findings, and the second-to-fourth digit (2D:4D) ratio in women with PCOS compared to age-matched healthy controls.

Methods

A case-control study was conducted, including women diagnosed with PCOS and age-matched healthy controls. Clinical features, anthropometric measurements (BMI, waist-hip ratio (WHR), waist-height ratio (WHtR)), hormonal parameters (luteinizing hormone (LH), follicle-stimulating hormone (FSH), anti-Müllerian hormone (AMH)), metabolic variables (fasting glucose, lipid profile), ultrasonographic findings, and 2D:4D digit ratio were assessed. Statistical analysis was performed using appropriate tests, and a p-value < 0.05 was considered statistically significant.

Results

Women with PCOS exhibited a significantly higher prevalence of menstrual irregularity, polycystic ovarian morphology, hirsutism, and acne (p < 0.001). Hormonal analysis showed significantly elevated LH, FSH, and AMH levels in PCOS cases (p < 0.001). Anthropometric indices, including BMI, WHR, and WHtR, were significantly higher among PCOS cases (p < 0.01), indicating increased general and central adiposity. In contrast, fasting glucose and lipid profile levels did not differ significantly between groups. Additionally, the 2D:4D ratio was significantly lower in PCOS cases (p < 0.01) and showed significant negative correlations with BMI, WHR, WHtR, and cholesterol levels.

Conclusion

The study demonstrates that PCOS is associated with significant hormonal dysregulation, central obesity, and early metabolic alterations. The lower 2D:4D ratio observed in PCOS supports the role of prenatal androgen exposure in disease pathogenesis. These findings highlight the complex interplay between developmental, metabolic, and endocrine factors in PCOS and underscore the importance of early identification and comprehensive management strategies.

## Introduction

Polycystic ovary syndrome (PCOS) is a heterogeneous endocrine and metabolic disorder that primarily affects women of reproductive age. It is characterized by a range of reproductive, metabolic, and psychological symptoms and impacts approximately 5%-20% of this population globally [1]. The hallmark features of PCOS include chronic anovulation, hyperandrogenism, and polycystic ovarian morphology on ultrasonography. Common clinical manifestations include irregular menstrual cycles, infertility, hirsutism, androgenic alopecia, weight gain, insulin resistance, and an elevated risk of developing type 2 diabetes mellitus [2,3]. In addition, psychological disturbances such as anxiety, stress, and reduced quality of life are frequently reported [4]. Given the multifactorial nature of PCOS, anthropometric measurements serve as simple, non-invasive, and cost-effective tools for assessing body composition, fat distribution, and associated metabolic risk.

Based on body composition and metabolic profiles, PCOS is broadly classified into distinct phenotypes: lean, normal-weight, and obese. Lean PCOS patients, despite having normal or low BMI, often exhibit significant hormonal disturbances such as elevated luteinizing hormone (LH) and anti-Müllerian hormone (AMH) levels, suggesting that PCOS is not solely an obesity-driven disorder. In contrast, obese PCOS is characterized by higher BMI, increased waist-hip and waist-height ratios, pronounced insulin resistance, dyslipidemia, and greater cardiometabolic risk [5-8]. These endocrine abnormalities interact closely with adiposity and insulin resistance, further aggravating metabolic and reproductive dysfunction. Normal-weight PCOS represents an intermediate phenotype with variable metabolic and endocrine features [9].

Body mass index (BMI) is the most commonly used anthropometric indicator for assessing overall adiposity and classifying individuals as lean, normal weight, overweight, or obese. Obesity, particularly central obesity, is highly prevalent in PCOS and is strongly associated with insulin resistance, hyperinsulinemia, and exacerbation of hyperandrogenism [10]. Elevated BMI in women with PCOS has been linked to worsening reproductive dysfunction, increased menstrual irregularities, and adverse metabolic outcomes [11]. However, BMI does not account for regional fat distribution, so additional anthropometric indices are needed.

The distribution of visceral fat, which is crucial for metabolic dysregulation, can be seen in the waist-hip ratio (WHR), a reliable measure of central or abdominal adiposity [12]. Women with PCOS often exhibit a higher WHR, indicating an android pattern of fat deposition. Increased WHR has been linked to insulin resistance, dyslipidemia, elevated lipid levels, inflammatory markers, and cardiovascular risk, even in women with normal BMI [12,13]. Thus, WHR provides valuable insight into metabolic risk beyond overall body weight.

Similarly, the waist-height ratio (WHtR) has emerged as a sensitive indicator of cardiometabolic risk and central obesity [14]. WHtR is considered superior to BMI in predicting insulin resistance and metabolic syndrome, as it accounts for individual height and fat distribution [15]. Elevated WHtR in PCOS has been linked to impaired glucose metabolism, dyslipidemia, and increased androgen levels, highlighting its utility in identifying high-risk individuals irrespective of BMI categories [15-17].

In addition to conventional anthropometric indices, the second-to-fourth digit ratio (2D:4D) has attracted attention as a potential biomarker of prenatal androgen exposure [18]. A lower 2D:4D ratio is thought to reflect higher in-utero androgen levels, which may predispose individuals to PCOS-related traits later in life. Emerging evidence suggests an association between altered 2D:4D ratios, hyperandrogenism, and metabolic abnormalities in PCOS, supporting the developmental origin hypothesis of the syndrome [19-22]. In this study, we aimed to investigate the association between anthropometric indices (BMI, waist-hip ratio, waist-height ratio, and 2D:4D digit ratio) and hormonal, metabolic, and lipid profile alterations in women with PCOS. Additionally, we sought to evaluate how these parameters differ between PCOS cases and controls and to explore their relevance in identifying PCOS phenotypes and associated metabolic risk.

## Materials and methods

This cross-sectional observational study included 102 women recruited from the Outpatient Department (OPD) of the Department of Obstetrics and Gynaecology at the Institute of Medical Sciences, Banaras Hindu University in Varanasi, India. This was a duration-based study (August 2024 to December 2025), and all eligible participants attending the outpatient department during the study period were included using consecutive sampling. The required sample size was estimated using the power-based sample size formula for comparing two independent means to ensure adequate statistical power for detecting significant differences between groups.

The required sample size was estimated using the standard formula for comparison of two independent means:

\[ n = \frac{2\,(Z_{\alpha/2} + Z_{\beta})^2 \,\sigma^2}{d^2} \]

where Z_α/2_ represents the standard normal deviate for the desired significance level, Z_β_ corresponds to the power of the study, σ is the pooled standard deviation, and d is the expected difference between the two group means.

The participants were divided into the PCOS group (n = 53) and the healthy control group (n = 49). The control group consisted of women with regular menstrual cycles, normal clinical examinations, and absence of polycystic ovarian morphology (PCOM) as confirmed by ultrasonography. All participants were recruited from the same geographic region.

The inclusion criteria for the PCOS group comprised women aged 18-30 years who met the diagnostic criteria outlined by the 2003 European Society of Human Reproduction and Embryology (ESHRE)/American Society for Reproductive Medicine (ASRM) (Rotterdam criteria) [23]. According to these criteria, PCOS is diagnosed when any two of the following three features are present: (i) oligo- or anovulation, (ii) clinical and/or biochemical signs of hyperandrogenism, and (iii) polycystic ovaries as seen on ultrasound (USG), after excluding other endocrine disorders. Exclusion criteria included known endocrine disorders such as thyroid dysfunction, hyperprolactinemia, malignancy, and recent use of hormonal medications.

Tools used for anthropometric measurements

Anthropometric measurements were obtained using standardized instruments and procedures to ensure accuracy and reproducibility.

Waist and hip circumference

A non-stretchable measuring tape was used to measure waist and hip circumferences.

Waist Circumference

Waist circumference was measured with the person standing upright, feet shoulder-width apart, and arms relaxed at the sides. The midpoint between the two anatomical landmarks, the top border of the iliac crest and the lower margin of the last palpable rib, was noted. At this halfway, the measuring tape was placed horizontally around the abdomen, making sure it was parallel to the floor and did not compress the skin. At the conclusion of a typical expiration, measurements were made.

Hip Circumference

Hip circumference was measured with the subject standing erect and arms by the sides. The measuring tape was placed around the widest portion of the buttocks, ensuring that it remained horizontal and snug without compressing the soft tissues.

Height Measurement

Height was measured using a portable stadiometer (IS IndoSurgicals, New Delhi, India). Participants were instructed to stand barefoot on a flat surface with their heels together and in contact with the vertical rod of the stadiometer. The heels, buttocks, and shoulder blades were pressed against the rod, while the head was aligned in the Frankfort horizontal plane. The sliding headpiece was gently lowered to rest on the crown of the head, compressing the hair. Height was recorded to the nearest 0.1 cm.

Weight Measurement

Body weight was measured using a calibrated digital weighing scale (AccuSure, New Delhi, India). The scale was placed on a firm, level surface. Participants were instructed to stand barefoot on the center of the scale with weight evenly distributed, and arms relaxed at the sides. Measurements were recorded to the nearest 0.1 kg, with participants standing still and without external support.

Measurement of 2D:4D Ratio

The second-to-fourth digit (2D:4D) ratio was measured using a precision Vernier caliper (Yuzuki, Mumbai, India). Measurements were obtained from the index finger (2D) and ring finger (4D) of the same hand. Participants were instructed to place their hand on a flat surface in a relaxed position, palm facing upward, without stretching or flexing the fingers.

The length of each digit was measured from the midpoint of the basal crease to the tip of the finger. The outer jaws of the Vernier caliper were positioned at the basal crease and extended to the distal tip to ensure accurate linear measurement. To enhance measurement reliability and minimize observer error, each digit was measured at least twice, and the average of the repeated measurements was used for analysis.

Biochemical and Hormonal Assessment

Venous blood samples were collected from all participants after an overnight fast. Serum samples were analyzed for hormonal, metabolic, and lipid parameters. The hormonal profile included follicle-stimulating hormone (FSH), luteinizing hormone (LH), and anti-Müllerian hormone (AMH). Metabolic assessment included fasting blood glucose levels. Lipid profile parameters included total cholesterol, high-density lipoprotein (HDL), and low-density lipoprotein (LDL). All biochemical analyses were performed using standard laboratory methods in the central clinical laboratory of the Institute of Medical Sciences, Banaras Hindu University.

Participant demographic, clinical, and lifestyle information was collected using a structured questionnaire administered during the outpatient visit. The questionnaire included information on age, menstrual history, symptoms of hyperandrogenism (hirsutism and acne), psychological symptoms (anxiety/stress), family history of diabetes, and place of residence. Clinical records and ultrasonography reports were also reviewed to confirm diagnostic features of polycystic ovary syndrome (PCOS).

In this study, PCOS status (PCOS cases vs healthy controls) was considered the primary independent variable. Anthropometric parameters including body mass index (BMI), waist-hip ratio (WHR), waist-height ratio (WHtR), and the second-to-fourth digit (2D:4D) ratio were also evaluated as independent variables to assess their association with hormonal and metabolic parameters.

The dependent variables included hormonal parameters (follicle-stimulating hormone (FSH), luteinizing hormone (LH), and anti-Müllerian hormone (AMH)) and metabolic parameters (fasting glucose, total cholesterol, high-density lipoprotein (HDL), and low-density lipoprotein (LDL)). Clinical characteristics such as menstrual irregularity, hirsutism, acne, and ultrasonographic features of polycystic ovarian morphology were also analyzed in relation to PCOS status.

Ethical approval

This is a cross-sectional observational study. The Research Ethics Committee of the Institute of Medical Sciences, Banaras Hindu University, Varanasi, India, approved the study (approval code: IMS/IEC/2024/7405, dated: 12-08-2024). Relevant guidelines and regulations are followed in all methods. Informed consent was obtained from all subjects and/or their legal guardians.

Statistical analysis

Statistical analysis was conducted using IBM SPSS Statistics version 26.0 year 2019 (IBM Corp., Armonk, NY, USA). Categorical data were analyzed with the chi-square test, while continuous data were compared between groups using an independent-samples t-test. Pearson's correlation coefficient was used to assess associations between variables. To visualize correlation patterns and relationships among variables, heatmaps and scatter plots were generated using R software version 4.5.1 (R Foundation for Statistical Computing, Vienna, Austria) year 2025. All tests were two-tailed, and a p-value of less than 0.05 was considered statistically significant.

## Results

Demographic and clinical characteristics of study participants

**Figure 1:**
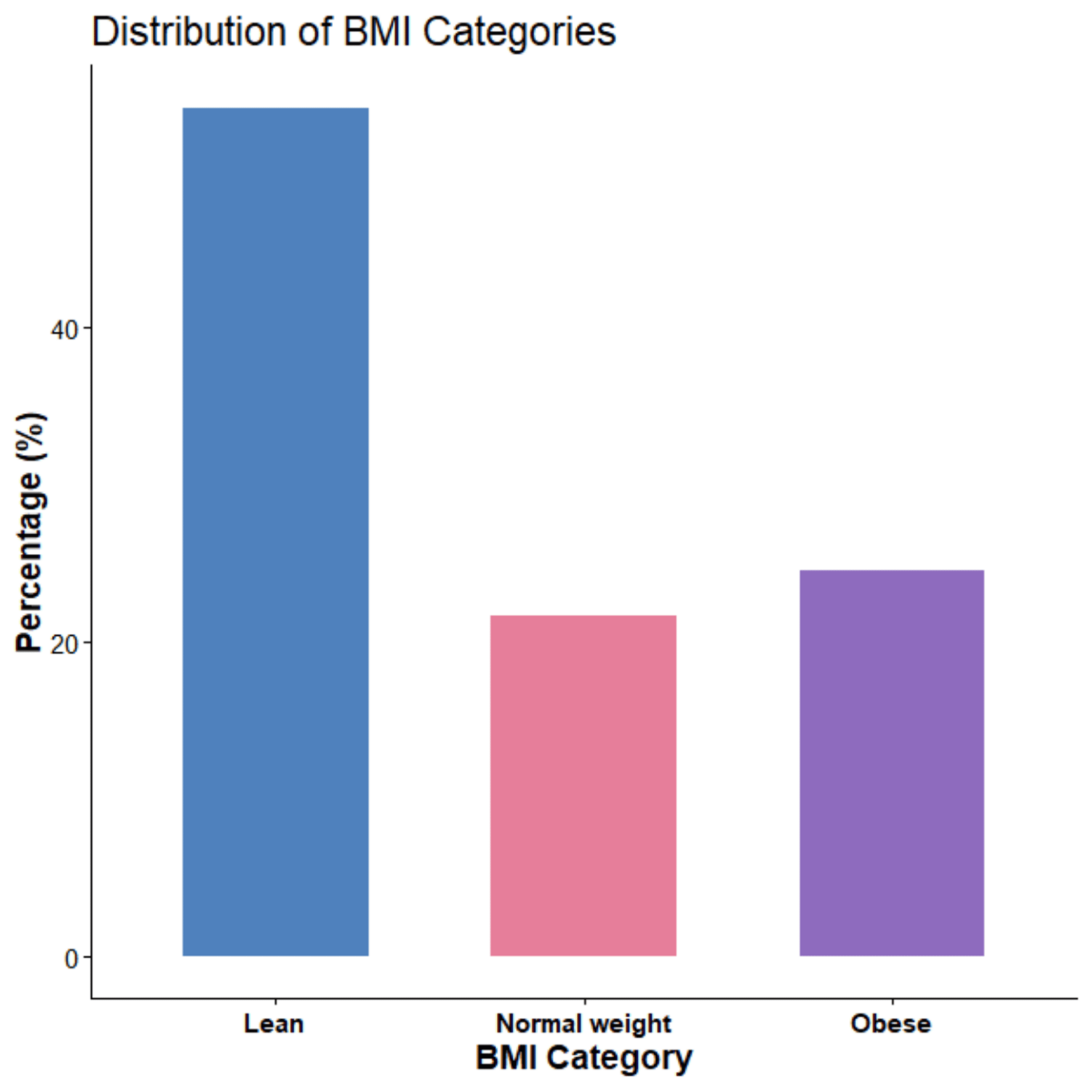
Distribution of BMI categories among the study participants.

The distribution of participants by BMI category is illustrated in the bar graph (Figure 1). Overall, lean individuals accounted for the most significant proportion of the study population (53.9%), followed by obese participants (24.5%) and normal-weight participants (21.6%). A comparative analysis of key clinical, lifestyle, and ultrasonographic variables between cases and controls was performed by chi-square test; the results are summarized below.

In Table 1, menstrual irregularity was observed in all PCOS cases (100%), whereas none of the controls reported irregular periods, showing a highly significant association with PCOS status (χ² = 102.000, p < 0.001). Ultrasonographic features suggestive of PCOS were present in 90.6% of cases, while all controls showed normal ovarian morphology, again demonstrating a considerable and statistically significant difference between groups (χ² = 83.824, p < 0.001).

**Table 1:** Clinical and demographic characteristics between the PCOS and the control. * indicates statistically significant differences between the groups, with p < 0.05. n (%) is a number of participants with percentage. χ², chi square; PCOS, polycystic ovary syndrome; USG: ultrasound.

Variable	Category	Cases (n=53) n (%)	Controls (n=49) n (%)	χ²	p-value
Irregular periods	Yes	53 (100.0)	0 (0.0)	102.000	<0.001*
	No	0 (0.0)	49 (100.0)		
Anxiety/Stress	Yes	26 (49.1)	17 (34.7)	2.154	0.142
	No	27 (50.9)	32 (65.3)		
Hirsutism	Yes	23 (43.4)	3 (6.1)	18.625	<0.001*
	No	30 (56.6)	46 (93.9)		
Acne	Yes	26 (49.1)	10 (20.4)	9.150	0.002*
	No	27 (50.9)	39 (79.6)		
Family diabetic history	Yes	23 (43.4)	13 (26.5)	3.171	0.075
	No	30 (56.6)	36 (73.5)		
Place of residence	Urban	25 (47.2)	33 (67.3)	4.226	0.040*
	Rural	28 (52.8)	16 (32.7)		
USG (PCOS features)	Present	48 (90.6)	0 (0.0)	83.824	<0.001*
	Absent	5 (9.4)	49 (100.0)		

Clinical hyperandrogenism, assessed through hirsutism and acne, was significantly more prevalent among PCOS cases. Hirsutism was reported in 43.4% of cases compared to only 6.1% of controls (χ² = 18.625, p < 0.001). Similarly, acne was present in 49.1% of cases and 20.4% of controls, indicating a significant association with PCOS (χ² = 9.150, p = 0.002).

Psychological symptoms such as anxiety and stress were more frequently reported among PCOS cases (49.1%) than controls (34.7%); however, this difference did not reach statistical significance (χ² = 2.154, p = 0.142). Likewise, a higher proportion of PCOS cases reported a family history of diabetes (43.4%) compared to controls (26.5%), although this association was not statistically significant (χ² = 3.171, p = 0.075). Analysis of the place of residence revealed a significant difference between groups. A greater proportion of PCOS cases were from rural areas (52.8%), whereas most controls resided in urban areas (67.3%) (χ² = 4.226, p = 0.040), suggesting a potential influence of residential or lifestyle factors on PCOS prevalence.

Comparison of anthropometric, hormonal, metabolic, and lipid parameters between groups

The comparison of continuous variables between PCOS cases and healthy controls is presented in Table 2. Age did not vary significantly between the two groups (21.58 ± 3.05 vs. 22.55 ± 2.62 years; p = 0.091), confirming appropriate age matching. Hormonal analysis revealed significant endocrine alterations in PCOS cases. Follicle-stimulating hormone (FSH), LH, and AMH levels were markedly higher in cases compared with controls (all p < 0.001), indicating disrupted gonadotropin regulation and increased ovarian reserve markers characteristic of PCOS.

**Table 2:** Biochemical and anthropometry parameters in PCOS and control groups. * indicates statistically significant differences between the groups, with p < 0.05. PCOS, polycystic ovary syndrome; SD, standard deviation; FSH, follicle-stimulating hormone; LH, luteinizing hormone; AMH, anti-Müllerian hormone; 2D:4D, second-to-fourth digit; LDL, low-density lipoprotein; HDL, high-density lipoprotein.

Variable	Cases (Mean ± SD)	Controls (Mean ± SD)	p-value
Age (years)	21.58 ± 3.05	22.55 ± 2.62	0.091
FSH (mIU/L)	6.02 ± 1.87	4.48 ± 0.71	<0.001*
LH (mIU/L)	11.85 ± 6.34	5.11 ± 0.80	<0.001*
AMH (ng/dL)	10.20 ± 4.76	4.43 ± 0.87	<0.001*
Fasting glucose (mg/dL)	81.65 ± 9.38	82.71 ± 7.70	0.536
BMI (kg/m²)	23.36 ± 4.80	20.73 ± 2.73	0.001*
Waist-hip ratio	0.793 ± 0.078	0.755 ± 0.055	0.005*
Waist-height ratio	0.197 ± 0.029	0.171 ± 0.020	<0.001*
2D:4D ratio	0.96 ± 0.22	0.97 ± 0.30	0.001*
Total cholesterol (mg/dL)	147.08 ± 25.16	144.18±15.05	0.487
HDL (mg/dL)	50.60 ± 8.94	48.98 ± 20.14	0.595
LDL (mg/dL)	78.85 ± 23.24	83.29 ± 25.46	0.360

Regarding anthropometric measures, BMI was significantly higher in PCOS cases (23.36 ± 4.80 kg/m²) than in controls (20.73 ± 2.73 kg/m²; p = 0.001). Indicators of central obesity, including waist-hip ratio and waist-height ratio, were significantly higher in the PCOS group (p = 0.005 and p < 0.001, respectively), reflecting greater abdominal adiposity.

Metabolic evaluation showed no significant difference in fasting glucose levels between cases and controls (p = 0.536). However, the 2D:4D ratio was significantly lower in PCOS cases than in controls (p = 0.001), suggesting a possible association with increased prenatal androgen exposure. Lipid profile assessment did not show a significant difference in total cholesterol levels between the groups (p = 0.487). Similarly, HDL and LDL concentrations also did not differ significantly between PCOS cases and controls (p = 0.595 and p = 0.360, respectively).

Correlation analysis and heatmap visualization of clinical and metabolic variables

**Figure 2:**
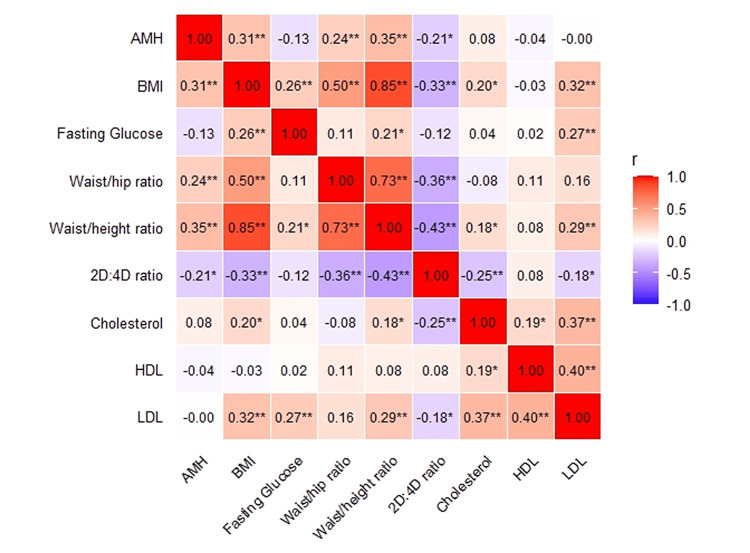
Heatmap showing Pearson correlation coefficients among anthropometric, hormonal, metabolic, and lipid profile variables. Red indicates positive correlations and blue indicates negative correlations, with color intensity proportional to the strength of correlation. Values represent Pearson’s r. AMH, anti-Müllerian hormone; 2D:4D, second-to-fourth digit ratio; HDL, high-density lipoprotein; LDL, low-density lipoprotein.

A Pearson correlation heatmap was generated to visualize the interrelationships among anthropometric, hormonal, metabolic, lipid, and digit ratio parameters (Figure 2). The heatmap shows several strong, statistically significant associations relevant to PCOS pathophysiology.

In Table 3, BMI showed a strong positive correlation with waist-height ratio (r = 0.850, p < 0.001) and waist-hip ratio (r = 0.504, p < 0.001), indicating that increased overall adiposity is closely linked to central obesity. BMI was also positively correlated with AMH (r = 0.308, p < 0.001) and total cholesterol (r = 0.402, p < 0.001), suggesting an association between adiposity, ovarian reserve markers, and dyslipidemia.

**Table 3:** Pearson correlation matrix of anthropometric, hormonal, metabolic, and lipid variables. * indicates p < 0.05 (statistically significant). ** indicates p < 0.01 (highly significant). Values represent Pearson’s correlation coefficients (r). AMH, anti-Müllerian hormone; 2D:4D, second-to-fourth digit; HDL, high-density lipoprotein; LDL, low-density lipoprotein.

Variable	AMH	BMI	Fasting Glucose	Waist/hip ratio	Waist/height ratio	2D:4D ratio	Cholesterol	HDL	LDL
AMH	1	.308**	-.131	.245**	.347**	-.211*	.085	-.040	-.004
BMI	.308**	1	.262**	.504**	.850**	-.327**	.198*	-.030	.319**
Fasting Glucose	-.131	.262**	1	.111	.206*	-.124	.043	.017	.267**
Waist/hip ratio	.245**	.504**	.111	1	.731**	-.356**	-.079	.107	.161
Waist/height ratio	.347**	.850**	.206*	.731**	1	-.430**	.179*	.080	.290**
2D:4D ratio	-.211*	-.327**	-.124	-.356**	-.430**	1	-.252**	.082	-.183*
Cholesterol	.085	.198*	.043	-.079	.179*	-.252**	1	.193*	.366**
HDL	-.040	-.030	.017	.107	.080	.082	.193*	1	.399**
LDL	-.004	.319**	.267**	.161	.290**	-.183*	.366**	.399**	1

Waist-hip ratio and waist-height ratio were strongly interrelated (r = 0.731, p < 0.001) and both demonstrated significant positive correlations with cholesterol levels, reflecting the metabolic risk associated with abdominal fat distribution. Notably, the 2D:4D ratio exhibited strong negative correlations with BMI (r = -0.724, p < 0.001), waist-height ratio (r = -0.599, p < 0.001), and waist-hip ratio (r = -0.304, p < 0.001), indicating that a lower digit ratio, suggestive of higher prenatal androgen exposure, is associated with increased adiposity and central obesity. Additionally, the 2D:4D ratio was negatively correlated with AMH and cholesterol levels.

AMH presented moderate positive correlations with cholesterol (r = 0.581, p < 0.001) and central obesity indices, reinforcing its link with metabolic disturbances in PCOS. Fasting glucose demonstrated weaker but significant associations with BMI, waist-height ratio, and LDL cholesterol. Overall, the heatmap highlights clustering among obesity indices, hormonal imbalance, lipid abnormalities, and digit ratio, underscoring the multifactorial metabolic-endocrine interactions underlying PCOS.

**Figure 3:**
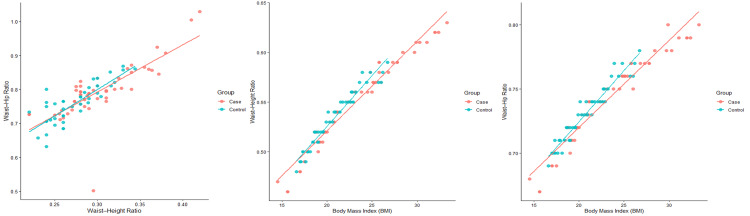
Scatter plots showing significant positive correlations between anthropometric indices in PCOS cases and controls: waist-hip ratio (WHR) vs waist-height ratio (WHtR) (r = 0.731, p < 0.01), body mass index (BMI) vs WHR (r = 0.504, p < 0.01), and BMI vs WHtR (r = 0.850, p < 0.01). PCOS: polycystic ovary syndrome.

Scatter plot analysis was performed to evaluate the linear relationships among waist-hip ratio (WHR), waist-height ratio (WHtR), and body mass index (BMI) in PCOS cases and controls (Figure 3). A strong positive correlation was observed between WHR and WHtR (r = 0.731, p < 0.001), indicating that increases in abdominal adiposity are consistently reflected across central obesity indices.

BMI presented a moderate positive correlation with WHR (r = 0.504, p < 0.001) and a robust positive correlation with WHtR (r = 0.850, p < 0.001), highlighting WHtR as a more sensitive marker of BMI-associated adiposity. In all three scatter plots, PCOS cases showed greater clustering toward higher BMI, WHR, and WHtR values than controls, with clear linear trends across groups.

## Discussion

Polycystic ovary syndrome (PCOS) is a heterogeneous endocrine-metabolic disorder characterized by reproductive, hormonal, and metabolic abnormalities. The present case-control study comprehensively evaluated clinical features, anthropometric indices, biochemical parameters, lipid profiles, and developmental markers in women with PCOS compared with age-matched healthy controls. Our findings provide important insights into the interrelationships between hyperandrogenism, adiposity, gonadotropin dysregulation, metabolic risk, and developmental influences in PCOS.

**Figure 4:**
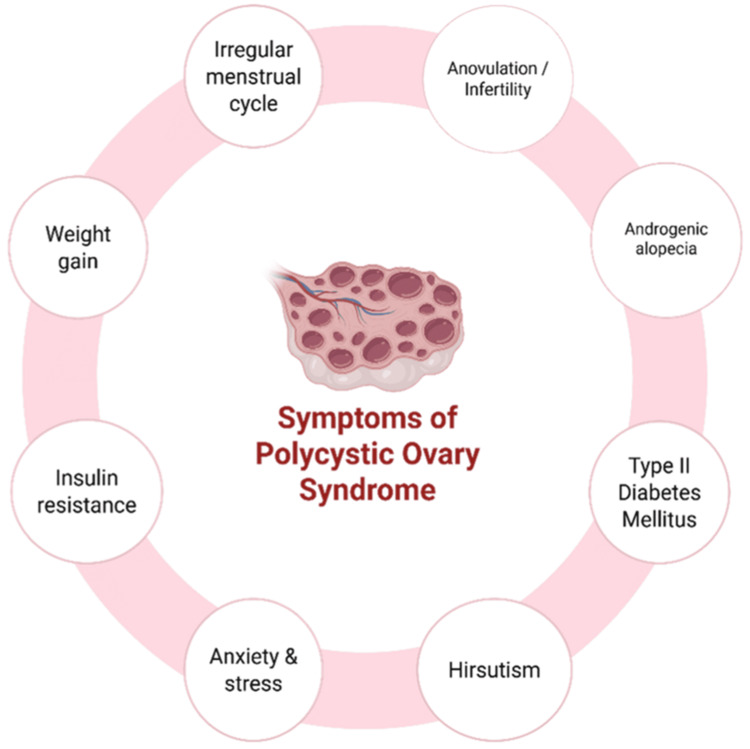
Clinical and metabolic symptoms of polycystic ovary syndrome (PCOS). Created by BioRender (Toronto, Canada).

Clinically, irregular menstrual cycles and ultrasonographic evidence of polycystic ovaries were observed almost exclusively among PCOS cases, confirming their diagnostic significance. The high prevalence of hirsutism and acne in cases further reflects the hyperandrogenic state characteristic of PCOS (Figure 4). These observations are consistent with established diagnostic criteria [23] and reinforce the role of androgen excess in driving both reproductive and dermatological manifestations of the disorder. Although anxiety and stress were more frequently reported among PCOS cases, the difference was not statistically significant, suggesting that additional social and environmental factors beyond endocrine dysfunction alone may modulate psychological symptoms.

Endocrine profiling revealed significantly elevated levels of luteinizing hormone (LH), follicle-stimulating hormone (FSH), and anti-Müllerian hormone (AMH) in women with PCOS. Increased LH levels support the concept of altered hypothalamic-pituitary-ovarian axis activity, potentially due to increased gonadotropin-releasing hormone (GnRH) pulse frequency, which preferentially stimulates LH secretion. Elevated AMH concentrations are indicative of increased antral follicle number and impaired follicular maturation, hallmarks of PCOS. Together, these hormonal abnormalities provide mechanistic insight into the observed anovulation and infertility associated with the syndrome.

These findings are consistent with earlier studies showing that central obesity, rather than generalized obesity, plays a pivotal role in the metabolic and endocrine disturbances observed in PCOS [24]. Lim et al. demonstrated that waist-based indices show stronger associations with insulin resistance and cardiovascular risk factors in women with PCOS than BMI alone [23]. Similarly, Dobbie et al. reported that increased abdominal adiposity amplifies hyperandrogenism and worsens reproductive outcomes, even in women who are not overtly obese [25].

Despite these endocrine disturbances, fasting glucose levels did not differ significantly between cases and controls, suggesting that overt glycemic dysregulation may not yet be apparent in this relatively young study population. However, anthropometric assessment revealed significantly higher body mass index (BMI), waist-hip ratio (WHR), and waist-height ratio (WHtR) among PCOS cases, indicating increased general and central adiposity. Central obesity is known to exacerbate insulin resistance and androgen excess, even in the absence of hyperglycemia, and may represent an early metabolic risk state in PCOS.

The distribution of BMI categories further demonstrated a higher proportion of overweight and obese individuals among PCOS cases compared with controls, as illustrated in the bar graph analysis. This finding emphasizes the close association between adiposity and PCOS severity and supports the concept that excess weight, particularly visceral fat accumulation, may aggravate both metabolic and reproductive abnormalities. Notably, WHtR showed the strongest association with BMI, underscoring its potential utility as a sensitive and practical marker of cardiometabolic risk in PCOS [26,27].

An important and novel aspect of the present study is the evaluation of the second-to-fourth digit (2D:4D) ratio, a putative biomarker of prenatal androgen exposure. Women with PCOS demonstrated a significantly lower 2D:4D ratio compared with controls, suggesting increased androgen exposure during fetal life. This observation supports the developmental origins hypothesis of PCOS, which proposes that prenatal androgen excess programs long-term alterations in endocrine and metabolic function, predisposing affected individuals to PCOS manifestations in adulthood. Consistent with our findings, previous studies have reported significantly lower 2D:4D ratios in women with PCOS compared with healthy controls, providing evidence that prenatal androgen exposure contributes to both reproductive and metabolic phenotypes of the syndrome.

Furthermore, the significant negative correlations observed between the 2D:4D ratio and BMI, waist-hip ratio (WHR), and waist-height ratio (WHtR) suggest that prenatal androgen exposure may influence adult body composition and central fat distribution. Similar associations between lower 2D:4D ratios and increased adiposity or metabolic risk have been documented in earlier population-based and PCOS-specific studies, supporting the biological plausibility of this relationship. These findings indicate that the 2D:4D ratio may serve as an indirect marker linking early hormonal milieu with later-life adiposity and metabolic risk [28,29].

Lipid profile analysis revealed significantly elevated total cholesterol levels in PCOS cases, while HDL and LDL concentrations did not vary significantly between groups. Previous studies have also demonstrated early dyslipidemia in young women with PCOS, particularly elevated total cholesterol, even in the absence of overt metabolic disease, suggesting that lipid abnormalities may develop early in the disease course. The lack of significant differences in HDL and LDL in the present study may be attributable to the relatively young age of participants and the limited duration of metabolic exposure [29].

The correlation heatmap further illustrated these complex interrelationships. BMI showed strong positive correlations with WHR and WHtR, confirming the internal consistency of the anthropometric measures. Women with PCOS exhibiting higher anthropometric indices such as body mass index and waist-hip ratio tend to show adverse adipokine profiles, which contribute to increased insulin resistance and predispose them to gestational diabetes mellitus [30]. Conversely, the 2D:4D ratio exhibited significant negative correlations with BMI, central obesity indices, and cholesterol, a pattern reported similarly in earlier studies examining androgen-driven metabolic risk, reinforcing its potential role as an inverse marker of androgen-related metabolic dysfunction in PCOS.

The present study has several strengths. First, it provides a comprehensive evaluation of anthropometric, hormonal, metabolic, and developmental parameters in women with PCOS within a single study design. Second, the inclusion of multiple anthropometric indices, including BMI, waist-hip ratio, and waist-height ratio, allowed for a more detailed assessment of both general and central adiposity. Third, the assessment of the second-to-fourth digit (2D:4D) ratio as a developmental marker of prenatal androgen exposure adds a novel dimension to the understanding of PCOS pathophysiology. Additionally, the inclusion of an age-matched healthy control group enabled meaningful comparisons between PCOS and non-PCOS participants, strengthening the validity of the findings.

This study has several limitations that should be considered when interpreting the findings. First, the sample size was relatively small and participants were recruited from a single tertiary care center, which may limit the generalizability of the results to broader populations. Second, the cross-sectional design of the study restricts the ability to establish causal relationships between anthropometric indices, hormonal alterations, metabolic parameters, and the 2D:4D digit ratio in PCOS. Additionally, the use of consecutive sampling from an outpatient department may introduce potential selection bias. Furthermore, the relatively young age of the participants may have masked the presence of long-term metabolic and cardiovascular complications associated with PCOS. Therefore, future studies involving larger, multicenter cohorts and longitudinal follow-up are needed to better understand the temporal relationships and long-term metabolic consequences of these associations.

## Conclusions

The study demonstrates a significant interplay between anthropometric, endocrine, and metabolic factors in women with PCOS, who showed higher BMI, central obesity indices, LH, AMH, and total cholesterol levels compared with controls, underscoring the coexistence of reproductive and metabolic dysfunction early in life and highlighting the contribution of both overall and central adiposity to the PCOS phenotype. A key finding was the significantly lower 2D:4D ratio in PCOS cases, supporting a developmental link to prenatal androgen exposure, with negative correlations between 2D:4D and anthropometric and metabolic parameters, indicating that early androgen programming may predispose to adverse fat distribution and metabolic risk independent of body weight. The associations among adiposity, AMH, and lipid abnormalities further suggest that central fat accumulation aggravates ovarian and metabolic dysfunction, emphasizing the value of combining anthropometric, hormonal, and developmental markers for early risk stratification and intervention in polycystic ovarian syndrome.
